# Super-resolution microscopy reveals structural diversity in molecular exchange among peptide amphiphile nanofibres

**DOI:** 10.1038/ncomms11561

**Published:** 2016-05-19

**Authors:** Ricardo M. P. da Silva, Daan van der Zwaag, Lorenzo Albertazzi, Sungsoo S. Lee, E. W. Meijer, Samuel I. Stupp

**Affiliations:** 1 Simpson Querrey Institute for BioNanotechnology (SQI), Northwestern University, Chicago, Illinois 60611, USA; 2 Laboratory of Macromolecular and Organic Chemistry and Institute for Complex Molecular Systems, Eindhoven University of Technology, Eindhoven MB 5600, The Netherlands; 3 Craniofacial Development & Stem Cell Biology, King's College London, London, SE1 9RT, UK; 4 Nanoscopy for Nanomedicine Group, Institute for Bioengineering of Catalonia (IBEC), Barcelona 08028, Spain; 5 Department of Materials Science and Engineering, Northwestern University, Evanston, Illinois 60208, USA; 6 Department of Chemistry, Northwestern University, Evanston, Illinois 60208, USA; 7 Department of Medicine, Northwestern University, Chicago, Illinois 60611, USA; 8 Department of Biomedical Engineering, Northwestern University, Evanston, Illinois 60208, USA

## Abstract

The dynamic behaviour of supramolecular systems is an important dimension of their potential functions. Here, we report on the use of stochastic optical reconstruction microscopy to study the molecular exchange of peptide amphiphile nanofibres, supramolecular systems known to have important biomedical functions. Solutions of nanofibres labelled with different dyes (Cy3 and Cy5) were mixed, and the distribution of dyes inserting into initially single-colour nanofibres was quantified using correlative image analysis. Our observations are consistent with an exchange mechanism involving monomers or small clusters of molecules inserting randomly into a fibre. Different exchange rates are observed within the same fibre, suggesting that local cohesive structures exist on the basis of β-sheet discontinuous domains. The results reported here show that peptide amphiphile supramolecular systems can be dynamic and that their intermolecular interactions affect exchange patterns. This information can be used to generate useful aggregate morphologies for improved biomedical function.

## Abstract (web summary)

Dynamic behaviour in supramolecular systems is an important aspect of their functionality. Here, the authors use stochastic optical reconstruction microscopy to unveil structural diversity in self-assembled peptide amphiphile nanofibres, with potential relevance to biomedical applications.

## Graphical abstract

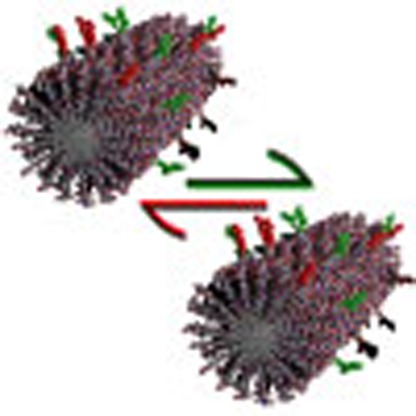

Reversible supramolecular interactions are ubiquitous in nature, controlling the self-assembly of ordered functional structures that need to be dynamic to perform their biological functions. One-dimensional cytoskeletal filaments such as actin and tubulin are typical examples of structures that use dynamics to mediate the adaptive behaviour of cells, resulting in cell motility, shape change, cell division, signalling and muscular contraction at larger length scales^1^,^2^,^3^,^4^,^5^,^6^. Artificial supramolecular materials could offer this bio-inspired dynamic behaviour, thus allowing enhanced interaction with natural systems and increased biomedical functionality. Since many natural processes are carefully regulated, optimization of an artificial supramolecular material requires a detailed understanding of its dynamic properties, for example its exchange kinetics.

Molecular mixing experiments typically assess exchange kinetics by utilizing Förster resonance energy transfer (FRET) between a pair of donor and acceptor fluorophores^7^,^8^, and alternatively, radio-labelled molecules^9^ or time-resolved small-angle neutron scattering^10^,^11^. Although these ensemble experiments can provide the timescale of the processes, they cannot distinguish different mechanisms. Moreover, they fail to detect the structural diversity among fibres, or within an individual fibre, for example, the occurrence of segregated domains. Local variations of molecular composition can have important biological implications, as they can greatly influence the signalling potency through multivalency effects^12^,^13^,^14^, and thus understanding the exchange heterogeneity is important to design materials in which function is connected with dynamics for adaptive or responsive behaviour^15^.

Super-resolution techniques are powerful tools to reveal the spatial distribution of molecules at the nanoscale, but these techniques have thus far been mainly applied to imaging fine details of cellular structures^16^,^17^. For instance, a resolution of ∼20 nm can be achieved using stochastic optical reconstruction microscopy (STORM)^18^, which is an order of magnitude below the diffraction limit and near the molecular scale. The enhanced resolution is achieved through the accurate localization of single fluorescent molecules; to identify individual fluorophores only a sparse subset of labels should be active at any given time. This sparse population of fluorophores is obtained using probes that can be photo-switched to a temporary non-fluorescent ‘off' state by light. By repeatedly activating different subsets and overlaying the resulting localizations, an image can be reconstructed. We have previously reported how to apply STORM to probe the dynamics of supramolecular fibres^19^. Using two-colour STORM and quantitative image analysis, we were able to resolve the monomer distribution along the fibre backbone. By following the monomer distribution during the molecular exchange process, we were able to infer the exchange mechanism.

Peptide amphiphiles (PAs) that self-assemble into high aspect ratio objects offer exciting opportunities for regenerative medicine and other therapeutic applications^20^,^21^,^22^,^23^,^24^. As illustrated in Fig. 1a, this class of molecules is composed of an unbranched alkyl chain linked to a peptide segment, which can be further subdivided in several domains. A segment with propensity to form β-sheets is conjugated to the alkyl tail, followed by a charged segment for solubility. Additional domains can be conjugated to the canonical structure to introduce biofunctionality in the nanofibres. Whereas the hydrophobic collapse of the aliphatic tails induces self-assembly, experimental^25^,^26^ and theoretical^27^ evidence suggests that the formation of directional hydrogen bonds within the β-sheet domain is an additional important component of the driving force for assembly of the molecules into one-dimensional filamentous shapes. The facile incorporation of multiple bioactive signals at controlled concentrations^14^,^28^, together with their structural resemblance with extracellular matrix fibres makes PA assemblies useful as bioactive artificial extracellular matrix components for cell signalling. Furthermore, they are also intrinsically biocompatible and biodegradable and can therefore disappear easily after fulfilling their biological functions. PAs have been extensively studied as a platform for applications that include bone, cartilage, enamel, neuronal regeneration, angiogenesis for ischaemic disease, targeted drug delivery and cancer therapeutics^20^,^21^,^22^,^23^,^24^,^29^.

Ensemble measurements of PA dynamics revealed partial self-healing using rheological techniques^30^ or the existence of a critical aggregation concentration^31^, while kinetic spectroscopy showed pathway selection of PAs into different morphologies^32^,^33^. However, these approaches do not consider structural heterogeneity and its effect on dynamic molecular exchange at the level of individual filaments. In this paper, we use STORM to image individual PA nanofibres, addressing the distribution of molecules along the fibre during exchange and therefore analysing diversity among supramolecular nanofibres.

## Results

### Nanofibre design and preparation

**Figure 1:**
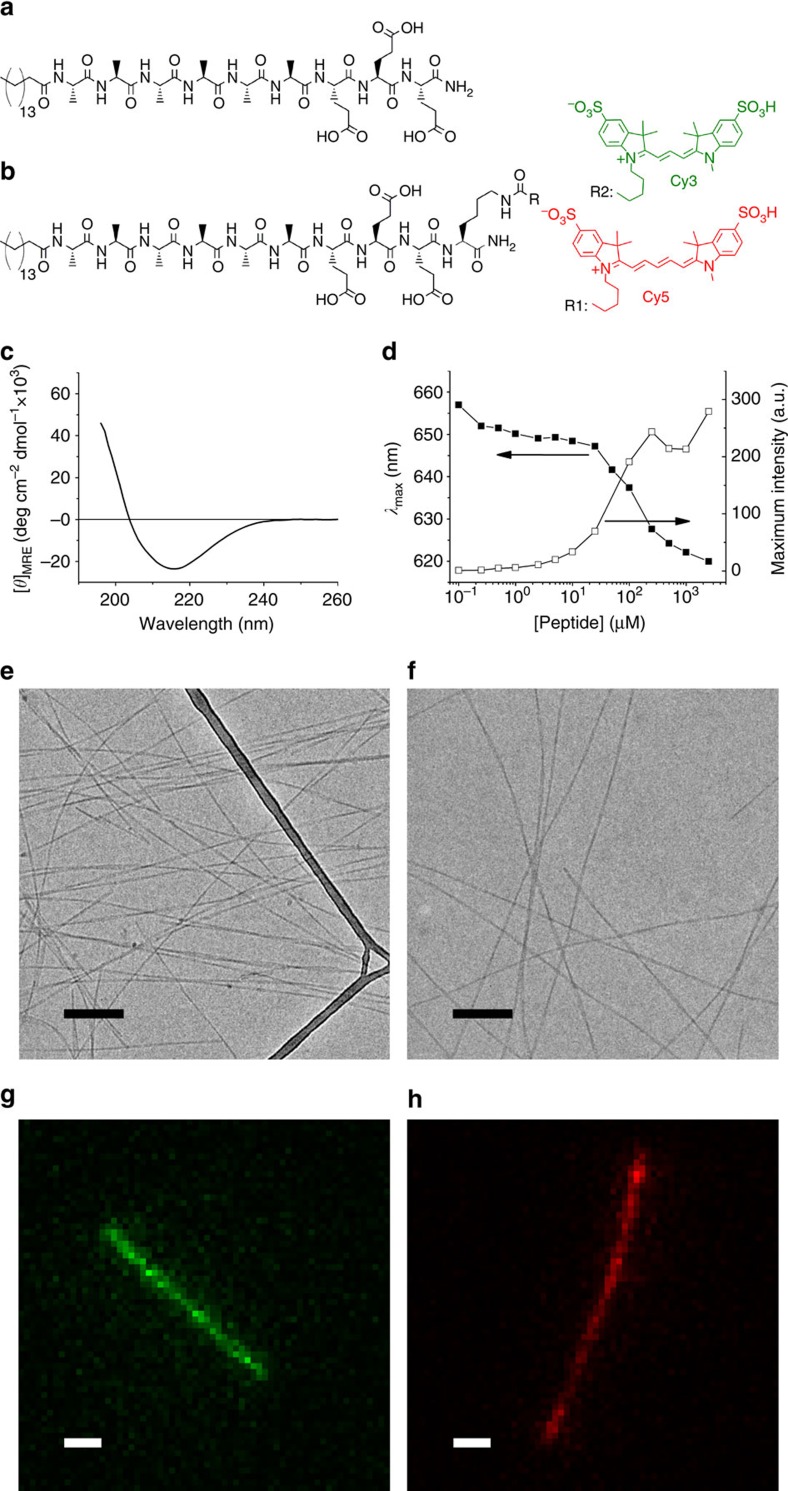
PA self-assembly. Molecular structure of (**a**) non-labelled PA and (**b**) PA molecules labelled with photo-switchable sulfonated cyanine dyes, namely Cy3 (green) and Cy5 (red). (**c**) Circular dichroism spectrum and (**d**) Nile Red assay of non-labelled PA. CryoTEM images of nanofibres self-assembled at pH 7.5 and NaCl 150 mM (**e**) from non-labelled PA alone and (**f**) from a molecular mixture of non-labelled and Cy5-labelled PAs (scale bar, 200 nm). Diffraction-limited fluorescence microscopy images of Cy3- (**g**) and Cy5-labelled (**h**) PA nanofibres (scale bar, 1 μm).

The PA molecule studied in this work is shown in Fig. 1a. It consists of a palmitic acid tail, six alanines in the β-sheet-forming region, followed by three glutamic acids as charged solubilizing moieties. This PA molecule self-assembles in water, forming nanofibres around 7 nm in diameter and lengths in the range of micrometres as observed by cryogenic transmission electron microscopy (cryoTEM; Fig. 1e). Amino acids with different propensities to form β-sheets affect considerably the internal order of PA assemblies, as well as nanofibre stiffness^26^. Since alanine has a weaker tendency to form β-sheets than valine^34^, placing this amino acid in the β-sheet-forming segment was expected to yield relatively dynamic PA fibres. Circular dichroism spectroscopy of this PA is consistent with the typical β-sheet conformation found for other PAs^26^, at physiological pH and ionic strength (Fig. 1c). To perform imaging by fluorescence microscopy, PA molecules were labelled with cyanine dyes, namely Cy3 and Cy5 (Fig. 1b). Water soluble sulfonated dyes with a net charge of −1 were used to preserve the amphiphilic asymmetry of the PA molecule and, since sulfonate groups are fully ionized in the vicinity of physiological pH, their behaviour is insensitive to pH in the range of interest. The Cy3 and Cy5 dyes have been chosen for their suitable photo-physical properties for STORM imaging; moreover, they constitute a good FRET pair with a Förster radius of 50 Å (ref. 35). Single-colour labelled nanofibres were created by mixing a stock solution of either Cy3-PA or Cy5-PA with a stock solution of unlabelled PA, lyophilization, brief co-dissolution in trifluoroacetic acid (TFA) and immediate TFA removal, lyophilization and final reconstitution in the aqueous working solution to form labelled supramolecular aggregates. The degree of labelling was accurately controlled by premixing the different PAs at the desired ratios. Fluorescently labelled nanofibres revealed a morphology that was indistinguishable from their non-labelled counterparts, as shown by cryoTEM (Fig. 1f).

### Ensemble molecular exchange kinetics

**Figure 2:**
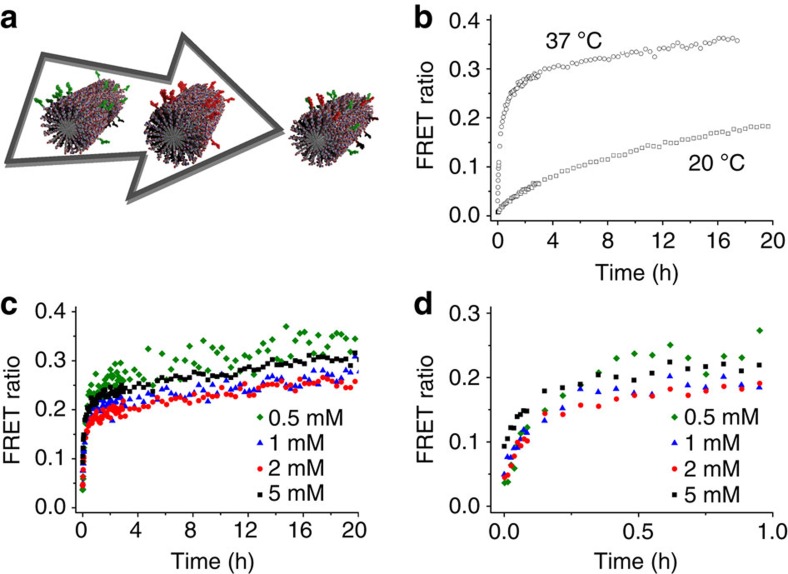
Molecular exchange by FRET. (**a**) Schematic representation of a molecular exchange kinetic measurement. The molecular exchange progress over time is estimated by means of FRET ratio (dividing Cy5 by Cy3 fluorescence intensities), either at (**b**) a constant PA concentration (0.5 mM) or at (**c**,**d**) a constant temperature (37 °C), where **d** shows the FRET ratio for short timescale.

The timescale of molecular exchange in PA-based nanofibres was measured using FRET kinetic experiments. Two sets of PA nanofibres were separately pre-assembled from a mixture of non-labelled PAs with either Cy3-PA (0.5%) or Cy5-PA (0.5%), as illustrated in Fig. 2a. Next, the two solutions were mixed and the FRET ratio, defined as the ratio between the fluorescence intensities of Cy5 acceptor and Cy3 donor, was monitored over time. As shown in Fig. 2b, the FRET ratio increases with time, reaching a plateau after several hours. This means that PA molecules are able to migrate between nanofibres, resulting in mixed fluorophore fibres. Figure 2b depicts kinetic experiments at different temperatures, showing that the exchange rate is remarkably faster at 37 °C than at 20 °C. On the other hand, we observed a limited effect of concentration on the exchange rate (Fig. 2c), proving that the exchange process is not diffusion limited in this concentration range. These results resemble observations on self-assembled polymeric micelles, in which unimer expulsion and insertion is thought to be the rate-determining step of system dynamics^10^,^11^, as well as other synthetic supramolecular polymers^7^. So, while FRET measurements provide useful information about the timescale of the exchange, FRET is an ensemble technique, and it does not provide the spatially resolved information required to elucidate the mechanism of exchange.

### Imaging PA nanofibres

**Figure 3:**
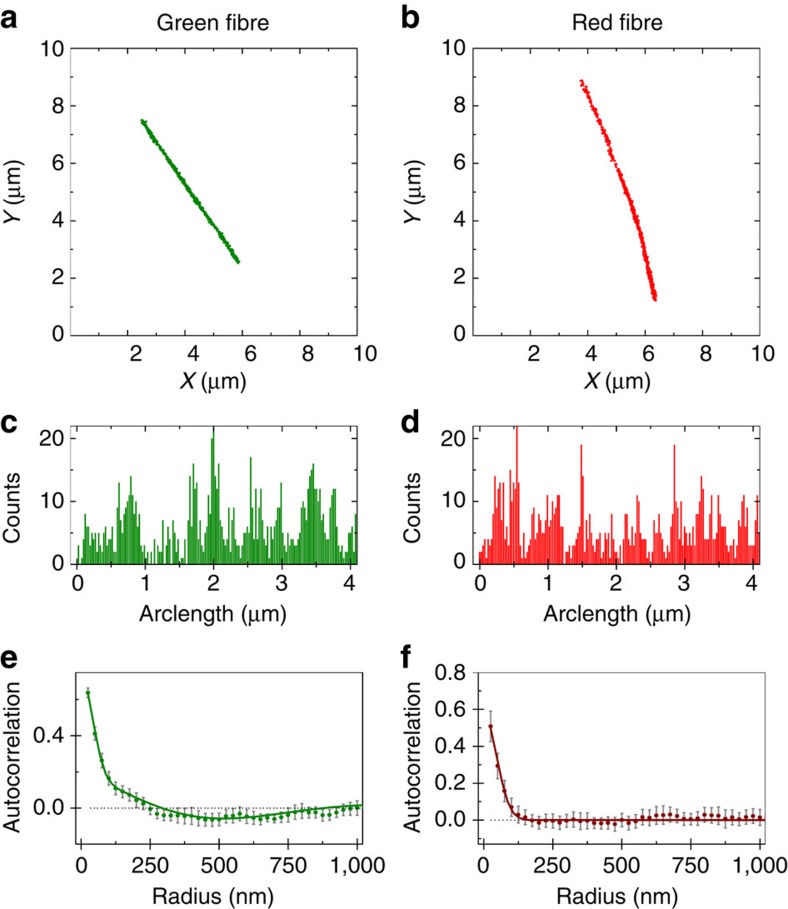
STORM analysis of single-colour nanofibres. PA nanofibres were labelled with 5 mol% of either Cy3-PA (**a**,**c**,**e**) or Cy5-PA (**b**,**d**,**f**) before the molecular exchange experiment. Localization maps after applying a clustering algorithm, background cleaning and backbone finding (**a**,**b**). Each fibre localization distribution profile was determined along the fibre backbone (arc length) using a bin size of 25 nm (**c**,**d**). Averaged autocorrelation (**e**,**f**) was computed from distribution profiles of a large set of fibre images (*n*⩾19). Solid lines correspond to model fittings (equation (1) for red channel and equation (2) for green channel) and error bars represent 95% confidence level.

Due to its high spatial resolution and multicolour imaging ability, STORM microscopy can be used to investigate the spatial details of the exchange process in PA nanofibres. Cy3- or Cy5-labelled PA nanofibres have been immobilized on a glass slide by physisorption and subsequently have been imaged using STORM (see the ‘Methods' Section for details). The resulting images show well-reconstructed aggregates (Fig. 3a,b), indicating that both dyes display appropriate photo-switching behaviour when associated to PA structures. Since the actual width of the nanofibre is below the STORM resolution, the apparent thickness of the fibre can be used to provide an estimate for the experimental resolution of these measurements, approximately equal to 50 nm (Supplementary Fig. 1). The fibre characteristics observed by STORM, for example, rigidity and fibre length, match those reported by the cryoTEM images in Fig. 1e,f.

To analyse the monomer distribution inside PA nanofibres, which is crucial to understand the exchange mechanism, we utilized an image analysis routine previously developed in the Meijer laboratory^19^. This method removes background localizations, identifies the contour of the fibre backbone to study its mechanical properties (Supplementary Fig. 2) and computes the localization density along the polymer. Figure 3c,d display the localization density plots for Cy5- and Cy3-labelled PA, respectively. These profiles contain information about the distribution of the dye-functionalized PA molecules in a nanofibre. As can be clearly observed, the number of localizations shows fluctuations along the fibre. These fluctuations can be attributed to two causes: (i) a heterogeneous distribution of monomers or (ii) the stochastic processes taking place during STORM image acquisition, for example, fluorophore blinking^36^ and fluorophore bleaching. Therefore, variations in the density of localizations cannot be unequivocally attributed to changes in local monomer concentration. To address this issue, we use a spatial autocorrelation algorithm, a powerful method to investigate the distribution of these localizations. In purely random distributions, localizations at distance *r* apart are not correlated with each other, by definition giving an autocorrelation *g*(*r*) with a value of zero. Positive values of *g*(*r*) represent an increased probability that a second localization is found at a certain distance (*r*) from a first given localization, while negative values indicate a decreased probability. The autocorrelation curves of fibres in single-colour samples are shown in Fig. 3e,f. The autocorrelation of Cy5-labelled nanofibres (Fig. 3f) is strongly positive in the short range (<100 nm) and zero for longer distances. This short-ranged contribution is the typical signature for multiple localizations of the same dye (overcounting) and is present in all STORM images. As previously described, the overcounting contribution in the spatial autocorrelation traces can be modelled using equation (1) (ref. 37),


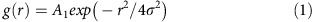


where the standard deviation (*σ*) provides an estimate of the resolution achieved. Equation (1) provides a good fit to the autocorrelations of Cy5-labelled single-colour nanofibres (Fig. 3f). Therefore, the localization density fluctuations visible in the STORM images for these PA nanofibres (Fig. 3d) are due to stochastic processes inherent to the technique, while the monomer distribution is homogeneous. On the other hand, the autocorrelation function for the Cy3-channel (Fig. 3e) is not well-described by Equation (1), since the Cy3-functionalized fibres display a consistent anti-correlation (*g*(*r*)<0) at intermediate distances (∼500 nm). The autocorrelation behaviour of these fibres was correctly described using a micro-emulsion model, that accounts for the existence of ‘microdomains' richer in the fluorescent monomer, according to equation (2) (ref. 37):


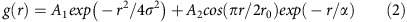


The first term of this equation is equal to equation (1), since the overcounting phenomenon is still present. In the second term, the parameter *r*_0_ is the average size of the ‘microdomains' and *α* is the coherence length of the domains. As defined by equation (2), ‘microdomain' corresponds to an extended fibre region that is enriched in fluorescently labelled molecules compared with the fibre average, thus increasing the likelihood of finding those molecules in that particular region. In this context, ‘microdomain' does not mean that molecules are completely clustered and confined to those regions.

An improvement in the goodness of the fit was not observed for the Cy5-labelled nanofibres, therefore it is reasonable to adopt the simpler model with less parameters, which excludes the existence of ‘microdomains'. On the other hand, in the case of Cy3-labelled nanofibres a considerable improvement on the *χ*^2^-test (>3.5 times lower) is obtained for the model with the ‘microdomain' component, compared with the model that only considers the contribution of the overcounting. A ‘microdomain' size of ∼300 nm and coherence length of ∼500 nm were obtained from the fit (Fig. 3e), showing a regular fluctuation of localization density in the Cy3-fibres at the submicron length scale. The difference observed between the distributions of Cy3 and Cy5-PAs is surprising, because of the great chemical and structural resemblance of these dyes. These results show that a small change in the PA structure may have noticeable effects on the self-assembly process, and that the STORM-based autocorrelation analysis can be used to investigate structure at the single-aggregate level. In addition, it shows that Cy5-functionalized PA is suitable for use in quantitative exchange experiments, since the Cy5 dye does not affect the molecular distribution of the PA monomers.

### Fibre bundling and multicolour imaging

**Figure 4:**
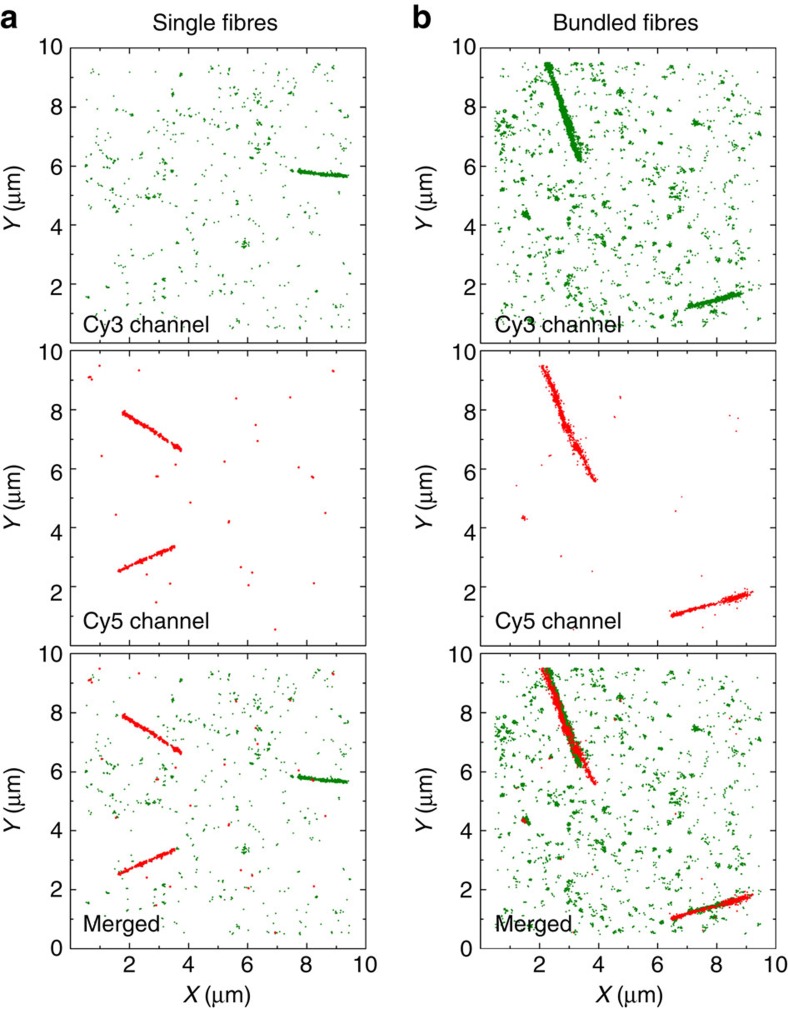
Single fibre attachment. STORM images (with background) of PA nanofibres attached at a final PA concentration of either (**a**) 0.1 μM or (**b**) 1 μM, showing single fibres or bundled fibres, respectively. Attachment was performed immediately on a glass coverslip after dilution (NaCl 1 M).

Since the final morphology in supramolecular polymers is highly sensitive to not only the molecular structure, but also the polymerization conditions, it is very important to design a correct sample preparation protocol to obtain the desired structure. A typical problem that may occur is the aggregation of fibres into bundles, a phenomenon that is hard to detect using traditional techniques. Avoiding fibre bundling is crucial when investigating molecular mixing, because bundles of nanofibres with different colours confound the observation of exchanging monomers. In this work we show that STORM-based analysis can be applied to detect PA fibre bundling. When nanofibre preparation was performed at higher PA concentration ([PA]_final_>1 μM), extremely long and curved fibrillar structures were observed in diffraction-limited fluorescence microscopy (Supplementary Fig. 3). However, the increased resolution attained using STORM imaging revealed finer details of the adsorbed structures, showing extensive fibre bundling. What appeared to be curvature at low resolution was actually the intersection of smaller stiff fibres with different orientations. Two-colour STORM has been performed on PA samples of different concentrations and ionic strengths to unambiguously prove the presence or absence of bundled fibres. Two aqueous solutions of PA nanofibres labelled with either Cy3 or Cy5 were mixed together and immediately adsorbed onto a glass coverslip at room temperature, freezing all kinetics. For this short mixing timescale (<1 min), molecular exchange is negligible according to the FRET measurements shown in Fig. 2b. Therefore, single fibres should be either fully green or fully red. If fibres are adsorbed at high ionic strength and low concentration (<0.1 μM), this behaviour was indeed observed (Fig. 4a). On the contrary, as shown in Fig. 4b, fibres adsorbed at concentrations higher than 1 μM can be observed in both channels simultaneously, indicating bundles of PA nanofibres. It is also possible to observe that some of the fibres visible in the green channel are not perfectly aligned with the overlapping fibres visible in the red channel (Fig. 4b). This provides further evidence that at this concentration bundles are observed instead of fully mixed fibres. Therefore, STORM allows us to verify the absence of fibre bundling and therefore select the optimal sample preparation to perform the molecular exchange experiments.

### Molecular exchange kinetics and mechanisms

**Figure 5:**
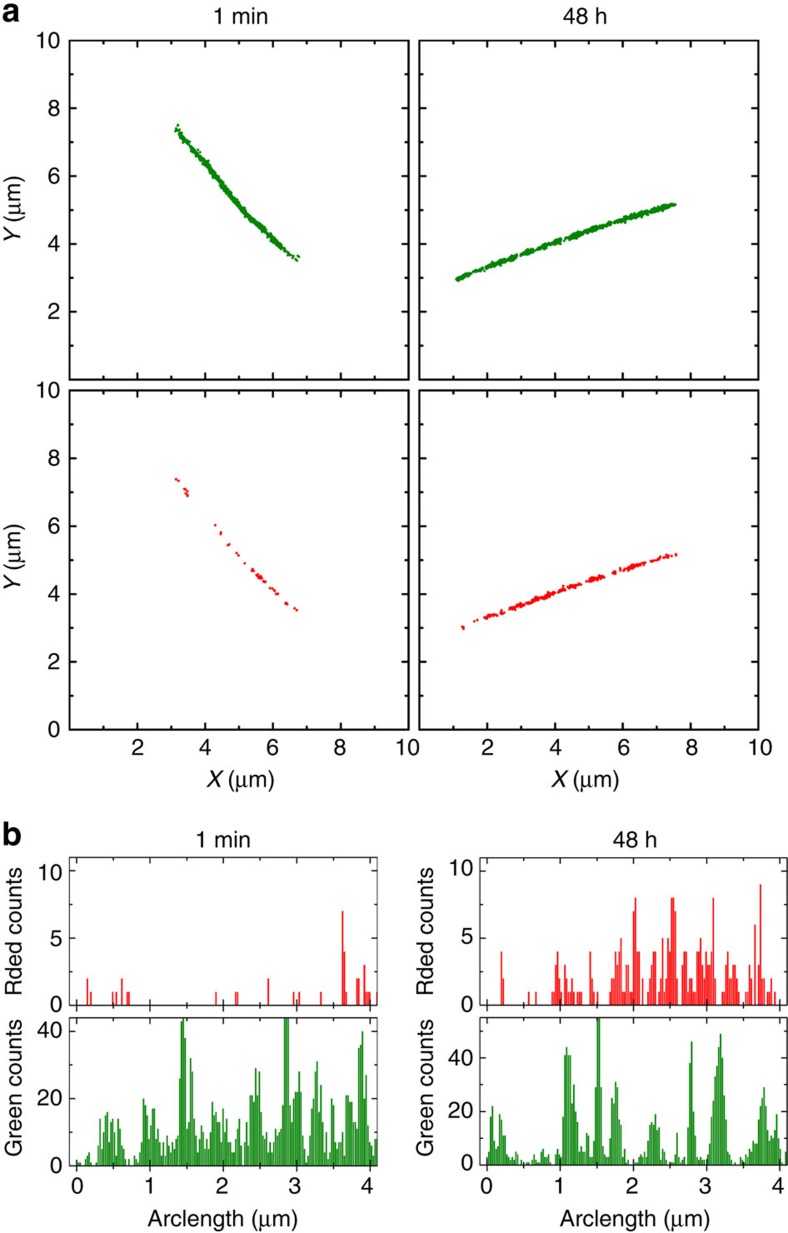
Quantitative analysis of STORM imaging during a molecular exchange experiment. (**a**) Localization maps of PA nanofibres immobilized on a glass coverslip at different time points, after applying a clustering algorithm, background cleaning and backbone finding. The better reconstruction of the fibres in the green channel reflects the selection of initially Cy3-labelled fibres for this experiment. (**b**) Histograms depict the localization density profiles along the nanofibre backbone (bin size 25 nm).

The ability of STORM to image individual nanofibres with high resolution and to study the distribution of different molecular species inside aggregates (*vide supra*), makes it the perfect tool to study exchange in PA samples. We prepared samples for these experiments in similar manner to the FRET measurements. First, two sets of single-colour PA nanofibres with either Cy3-PA (5%) or Cy5-PA (5%) were separately preassembled in aqueous buffer solution, followed by a 16-h aging period. Subsequently the two aged solutions were brought to 37 °C and mixed, allowing molecular exchange. Aliquots of the solution were withdrawn over the course of 48 h and nanofibres were adsorbed onto a glass coverslip at room temperature to freeze the exchange. Two-colour STORM images of assemblies were then acquired at the different time points. Fig. 5 shows representative PA nanofibres at *t*=1 min and after 48 h of mixing; as clearly shown in the images and in the corresponding localization density plot (Fig. 5b) the fibres initially containing a single label fully mix during the course of the experiment.

We verified that the exchange process is bidirectional by following green fibres incorporating red monomers (Supplementary Fig. 4) and vice versa (Supplementary Fig. 5). The intermediate time points provide insight into the exchange mechanism. Visual inspection of STORM images (Supplementary Fig. 6) showed insertion of labelled monomers over the entire length of the nanofibres throughout the exchange experiment. This finding suggests an exchange mechanism on the basis of expulsion and reinclusion of PA molecules. Polymerization–depolymerization at the nanofibre ends would have resulted in preferential exchange in those regions, while fragmentation–recombination would have resulted in a block-like structure; neither of these mechanisms is consistent with the acquired STORM images.

**Figure 6:**
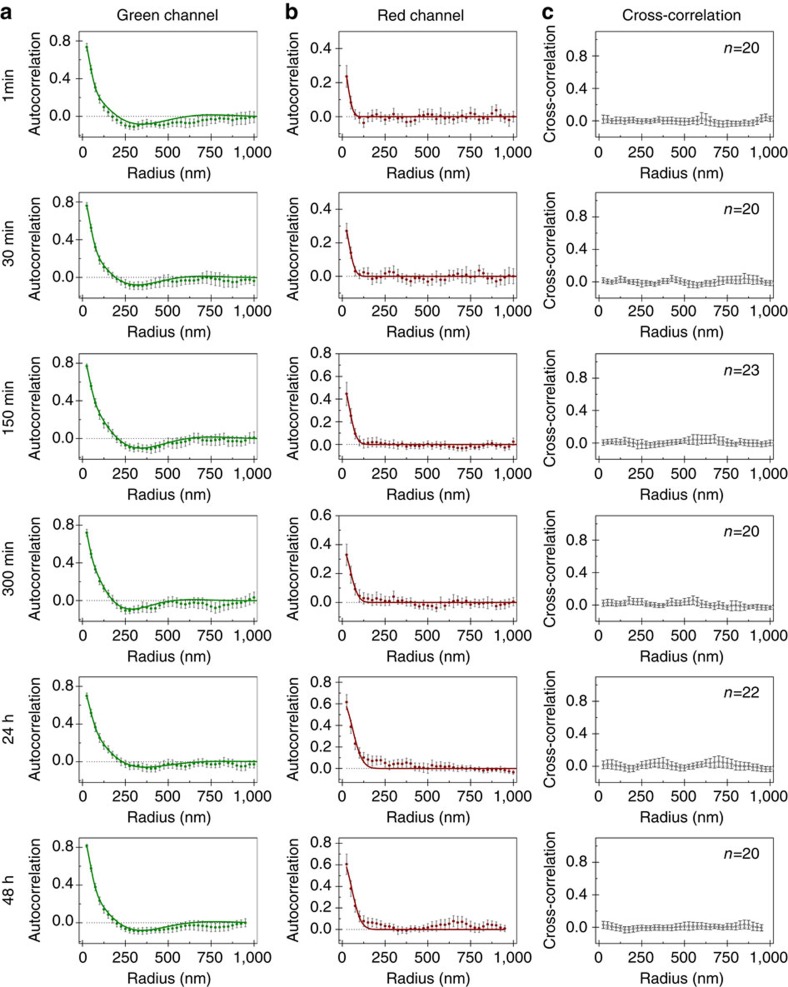
Correlation of cyanine dyes along PA nanofibres initially only labelled with Cy3 during molecular exchange. Averaged autocorrelation plots for (**a**) Cy3 and (**b**) Cy5-labelled PAs and (**c**) averaged cross-correlation between both dyes. Solid lines correspond to model fittings, namely equation (2) for (**a**) green channel and equation (1) for (**b**) red channel (error bars represent 95% confidence level, *n*⩾20).

To confirm this observation and further quantify the exchange, we studied the change in distribution of both dyes with time in initially green-labelled nanofibres, thus tracking the insertion of Cy5-PA. Figure 6 shows the auto- and cross-correlation plots for the green and red channels at the different time points. The green channel displays the previously observed clustered distribution described by equation (2) rendering it less amenable for quantitative analysis. Therefore, we monitored the autocorrelation of the red channel in time to analyse monomer exchange. The red channel autocorrelation, shown in Fig. 6b, can be fit with equation (1) for all time points, suggesting a random exchange of monomers. However, for later time points (that is, after incubation longer than 6 h) the quality of the fit deteriorates, indicating some non-random variations of the Cy5-PA concentration in the nanofibres other than the single decay originated from overcounting. However, the formation of regular ‘microdomains' does not seem plausible, because equation (2) did not improve the quality of the fit or yield reasonable fitting parameter values. Since clustering is not observed in single-colour Cy5-PA nanofibres (Fig. 3f), this observation points to a heterogeneous exchange and indicates the presence of structural variations along the fibre or between fibres. This heterogeneity can also be perceived visually by observing single fibre images (Supplementary Fig. 6). The cross-correlation between both channels was computed to assess how the distribution of one labelled PA would affect the distribution of the other, as shown in Fig. 6c. A cross-correlation of zero was found for the entire range, indicating that the distribution of Cy5-PA is not influenced by the presence of Cy3-PA, and vice versa. In other words, the heterogeneous exchange of Cy5-PA is not due to the pre-existing clusters of Cy3-PA, but rather a consequence of structural variation in the PA nanofibre.

**Figure 7:**
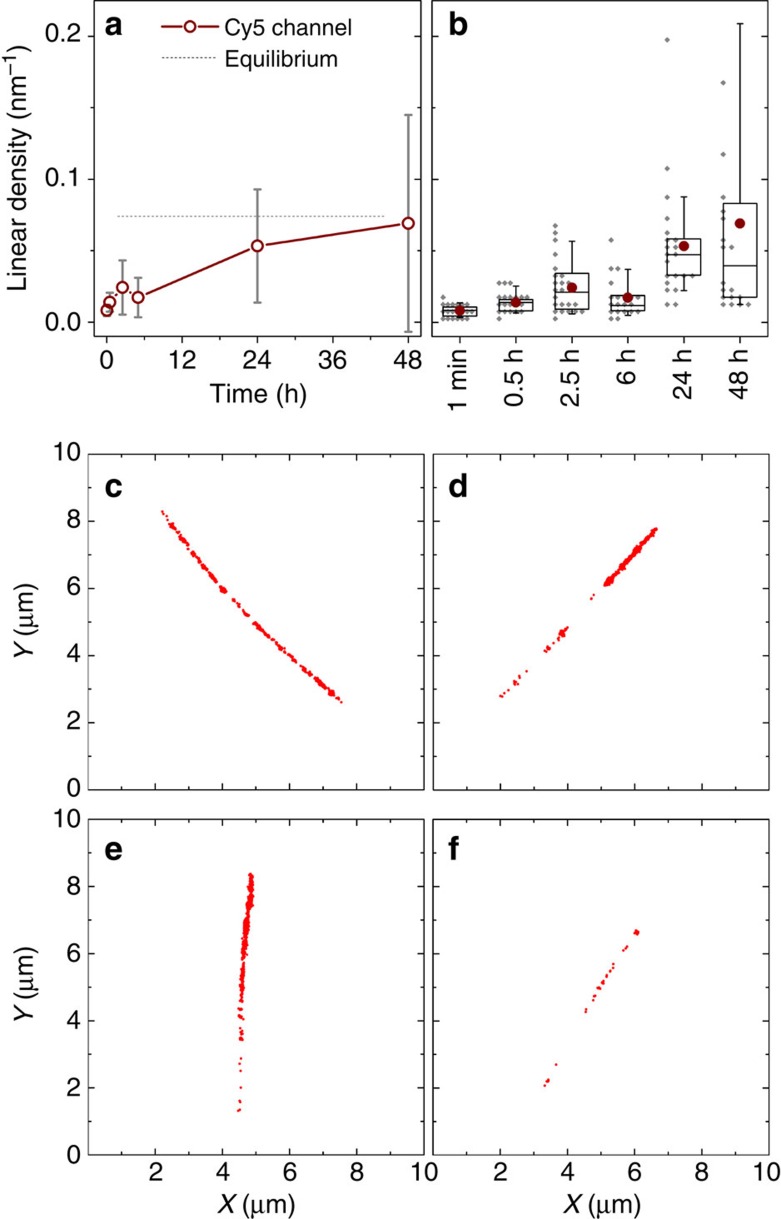
PA molecular exchange kinetics estimated using the linear density of localizations calculated from STORM data. (**a**) Time course-averaged density of red dye (Cy5) on fibres that were initially only labelled with green dyes (Cy3) (*n*⩾20, error bars correspond to standard deviation). The equilibrium guideline is estimated from the localization density of original single-colour red fibres. (**b**) Box chart of data represented on **a** depicting average (circle), median (box middle line), quartiles (box edges), 5 and 95% percentiles (error bars) and full data set points (diamond symbols, bin size 5 μm^−1^); (**c**–**f**) Examples of localization plots (after background cleaning) of individual fibres at 48 h.

The observation of this heterogeneity in exchange, not detected by the FRET experiment, was possible due to the ability of STORM to evaluate the progress of molecular exchange for each individual supramolecular nanofibre. The variability of the exchange progress between different aggregates could be measured, thus allowing us to further probe the heterogeneity in the system during and after molecular exchange. The linear density of localizations, defined as the number of localizations per nanometre arc length, has been computed and could be used as a rough estimate for the ensemble concentration of labelled molecules^19^. The density of Cy5-PA in originally single-colour green fibres was measured as a function of time, showing an increase in the average value as molecular exchange proceeded (Fig. 7a). However, the standard deviation also increased markedly, another indication for the presence of intrinsic structural diversity. In Fig. 7b, it is possible to observe how the distribution of the Cy5-PA linear density gets progressively wider as molecular exchange evolves in time. This can be confirmed visually by inspecting STORM images acquired after 48 h mixing time (Fig. 7c-f), which displayed a range of different behaviours. It was possible to observe a large subset of fibres that had a Cy5-PA density consistent with full mixing (Fig. 7c), as well as fibres comprised of domains of Cy3-PA only (Fig. 7d,e), and both subsets co-existed with a smaller subpopulation of nanofibres that had undergone very little exchange all over their length (Fig. 7f). The existence of regions displaying minimal or no exchange after 48 h implies that full equilibration of these regions will take weeks to months, making them persistent for most practical purposes. This analysis suggests that the population of fibres is considerably more heterogeneous after 48 h than would be expected on the basis of the FRET ensemble measurements.

Previous molecular dynamics simulations of a similar PA provide a theoretical framework for this structural diversity^27^. In these simulations, a broad distribution of secondary structures was found in the equilibrated fibre. The heterogeneous molecular exchange pattern observed in our study might stem from this conformational diversity. The key feature that led to the discovery of PA supramolecular nanofibres in the Stupp laboratory was the use of a β-sheet peptide domain to drive one-dimensional self-assembly. Using electron paramagnetic resonance, the β-sheet domain has been recently shown to give rise to locally solid-like behaviour in the interior of nanofibre PA assemblies^32^. On the other hand, the surface moieties in these nanofibres have been identified by the electron paramagnetic resonance experiments as regions where liquid-like dynamics prevail. β-Sheets within the nanofibres are highly cohesive assemblies and therefore the dynamic exchange among supramolecular nanofibres should give rise to a large diversity of supramolecular environments. This is in contrast to supramolecular systems with weaker internal cohesion that could exchange molecules to produce completely homogeneous environments^19^. The formulation of supramolecular systems with high levels of internal order and cohesion such as the PA nanofibres will therefore open new avenues to generate structural diversity for functional purposes. One example will be their ability to adapt structurally to bind important bioactive targets.

## Discussion

We have studied the dynamics of PA nanofibres using FRET and super-resolution localization microscopy. While ensemble FRET measurements prove that PAs exchange between different fibres, two-colour STORM imaging reveals a mechanism on the basis of the transfer of monomers and small clusters. Remarkably, the coexistence of fully dynamic and kinetically inactive areas in the aggregate architecture was observed, demonstrating the existence of structural diversity in PA nanofibres. This intriguing dynamic behaviour is foreseen to have important implications in the biological performance of supramolecular systems.

## Methods

### Peptide synthesis and purification

PAs were synthesized using standard Fmoc solid-phase peptide synthesis. MBHA rink amide resin, Fmoc-protected amino acids and other solid-phase peptide synthesis reagents were purchased from Novabiochem (USA). Cyanine dyes were supplied by Cyandye (USA). ACS-grade solvents were used for synthesis. Water was purified on an EMD Milipore Milli-Q Integral Water Purification System. For each amino acid coupling, 4.1 equiv of Fmoc-protected amino acid was activated for 1 min with 4 equiv of HBTU (O-benzotriazole-*N*,*N*,*N*′,*N*′-tetramethyluroniumhexafluorophosphate) and 6 equiv DIPEA (*N*,*N*-diisopropylethylamine) in 20 ml DMF. The coupling cocktail was then added to 0.5 mmol of MBHA rink resin and reacted for 1 h. Fmoc removal was performed with 30% piperidine in DMF. Fmoc-Glu(OtBu)-OH (N-α-Fmoc-L-glutamic acid γ-t-butyl ester) (3 × ), and Fmoc-Ala-OH (N-α-Fmoc-L-alanine) (6 × ) were successively coupled to the resin. Finally, the palmitic acid tail (8.1 equiv) was coupled using 8 equiv of HBTU and 12 equiv DIPEA in DMF/DCM (50:50). PAs were cleaved from the resin using a cleavage solution of 95% TFA, 2.5% TIPS (triisopropylsilane) and 2.5% H_2_O. Cleavage solution was concentrated by rotary evaporation and the PA precipitated in cold diethyl ether. The crude solid was dissolved in a diluted NH_4_OH aqueous solution (∼10 mg ml^−1^) and purified by preparative-scale reversed-phase HPLC on a Varian Prostar 210 HPLC system, using acetonitrile/water gradient containing 0.1% NH_4_OH. All eluents and additives were HPLC grade. Separation was achieved using a Phenomenex C18 Gemini NX column (150 × 30 mm) with 5 μm particle size and 110 Å pore size. Product-containing fractions were confirmed by ESI mass spectrometry (Agilent 6510 Q-TOF LC/MS) and combined. ACN was removed by rotary evaporation, lyophilized to yield a white solid product and stored at −30 °C.

### Cyanine dye coupling

PAs labelled with cyanine dyes were synthesized first coupling Fmoc-Lys(Mtt)-OH (*N*-α-Fmoc-*N*-ɛ-4-methyltrityl-L-lysine) to the resin. The rest of the synthetic procedure proceeded as described above. After coupling the palmitic acid tail, Lys(Mtt) was selectively deprotected with 3% TFA, 5% TIPS and 92% DCM for 5 min (3 × ), to expose the side chain amine moiety. Disulfo-cyanine carboxylic acid dyes (Cy3 and Cy5) were dissolved in DMF and stock solutions were stored at -30 °C. (Benzotriazol-1-yloxy)tripyrrolidinophosphonium hexafluorophosphate (PyBOP) was also dissolved in DMF. Cyanine dyes (1 equiv) were activated with PyBOP (1 equiv) and DIPEA (2 equiv) for 1 min and coupled to the lysine-free amine. Cy3- and Cy5-labelled PAs were cleaved and purified as described above. Lyophilization yielded a red powder for Cy3 and blue for Cy5.

### Purity

Purity was determined by reversed-phase analytical HPLC (see Supplementary Figs 7–9) using a Phenomenex Gemini C18 column (100 × 4.6 mm), with 5 μm particle size and 110 Å pore size, connected to a HPLC system equipped with an autosampler (Shimadzu SIL-20A XR), degasser (Shimadzu DGU-20A3) and a high-pressure gradient system comprising two LC-20AD XR pumps (Shimadzu). Separation was performed at a flow of 1 ml min^−1^ and using a 20-60% acetonitrile linear gradient in water containing 0.1% NH_4_OH. All eluents and additives were HPLC grade. Peptides, cyanine dye-coupled peptides and eventual contaminants were detected using a PDA (Shimadzu SPD-M20A), acquiring a full ultraviolet-visible spectrum between 200 and 750 nm at any time point. Main chromatogram peaks were collected and product mass confirmed by ESI mass spectrometry using a LCQ Deca XP Max (Thermo Finnigan) ion-trap mass spectrometer. For that, samples were manually injected bypassing the column at a flow rate of 0.20 ml min^−1^ and positive ion mass spectra were acquired in standard enhanced mode.

### Sample preparation

Stock solutions of non-labelled PA (10 mM), Cy3-labelled PA (1 mM) and Cy5-labelled PA (1 mM) were prepared in Milli-Q water, aliquoted and stored at −30 °C. Similar to other anionic PA systems, these formed hydrogels in the presence of CaCl_2_. The degree of labelling of the fibres was accurately controlled by simply mixing PA stock solutions at the desired ratio. Solutions of non-labelled PA were mixed with aqueous solutions of either one or both labels at certain mole ratios, flash-frozen in liquid nitrogen and lyophilized. The obtained PA mixture was molecularly dissolved in TFA and readily evaporated under vacuum. The obtained material was redissolved in NH_4_OH (aq.), flash-frozen and further lyophilized. These two last steps resembled the cleavage and purification conditions, respectively. These steps were undertaken to provide molecular mixing, as well as to reset PA self-assembly history. Non-labelled PA was treated using the same method before measurements where the fluorescence-labelled molecules were absent. The obtained powders were reconstituted in appropriate buffers before measurements. After the harsh acidic conditions (dissolution in TFA) used to create a homogenous molecular mixture, PAs were checked by analytical HPLC to rule out potential degradation of the original molecules. Analytical HPLC was performed as described above. Supplementary Fig. 10 shows that the PA does not degrade during the harsh procedure of molecular mixing in TFA. Fluorescence measurements showed pronounced FRET for nanofibres labelled with both Cy3 and Cy5. The FRET intensity increased with concentration of acceptor. This indicates that the sample preparation is suitable to assure co-assembly of the three PAs in a single supramolecular object.

### Circular dichroism spectroscopy

Circular dichroism spectra were recorded using Jasco Circular Dichroism Spectrometer (model J-715). PA was dissolved in water at 10 mM. Before each measurement, PA stock solution was diluted in appropriate buffers to yield a final PA concentration of 0.1 mM. A quartz cuvette of 1 mm path length was used for the measurements. Each trace represents the average of five scans.

### Cryogenic transmission electron microscopy

PA nanofibres were separately labelled with either PA-Cy3 (5%) or PA-Cy5 (5%). Both labelled and non-labelled PA nanofibres were dissolved at 1 mg ml^−1^ in 10 mM HEPES buffer (pH 7.5, NaCl 150 mM). CryoTEM was performed using a JEOL 1230 TEM at an accelerating voltage of 100 kV. Using a Vitrobot Mark IV (FEI) vitrification instrument at 25 °C with 100% humidity, a 6.5-μl drop of the sample was deposited on a 300-mesh copper grid with lacey carbon support (Electron Microscopy Sciences, EMS), blotted twice, plunge-frozen in liquid ethane, then stored in liquid N_2_. For imaging, the sample was transferred to a Gatan 626 cryo-holder under liquid N_2_, and images were obtained with a Gatan 831 CCD camera.

### Critical micelle concentration by Nile Red assay

Nile Red is a hydrophobic solvatochromic dye (water soluble at low concentration) with high affinity to the hydrophobic pocket of micelles. In aqueous environment, the molecule is weakly fluorescent. In hydrophobic environments, fluorescence intensity is around 300 times higher and a pronounced blue shift is observed. Therefore, Nile Red is commonly used to probe the existence of hydrophobic pockets formed by surfactants in aqueous environment^38^. Briefly, a Nile Red 2 mM stock solution was prepared in MeOH. Non-labelled PA was dissolved at 10 mM in 10 mM HEPES buffer (pH 7.5, NaCl 150 mM). PA solution was diluted serially to obtain concentrations in the range 200 nM-10 mM. Nile Red stock solution was diluted in the same buffer to obtain a final concentration of 1 μM. Equal volumes of Nile Red aqueous diluted solution and PA solutions were mixed for each PA concentration. Solutions were aged for 24 h to assure full nanofibre disassembly at concentrations below the critical micelle concentration. Fluorescence-emission spectra (excitation 550 nm) were recorded for an emission range between 570 and 700 nm. The maximum intensity and respective wavelength at maximum intensity were both represented as a function of the logarithm of the PA concentration. At concentrations close to critical micelle concentration, it should be observed a sharp increase in fluorescence intensity and a hypsochromic effect.

### Förster resonance energy transfer

Single-colour PA nanofibres labelled with either Cy3 or Cy5 at 1 mol% were assembled in 10 mM HEPES buffer (pH 7.5, NaCl 150 mM). Fluorescence spectra were recorded in a Varian Cary Eclipse Fluorimeter from Agilent Technologies. Emission spectra were acquired using excitation wavelengths of 520 or 615 nm for Cy3 or Cy5, respectively. Excitation spectra were collected using emission wavelengths of 600 or 710 nm for Cy3 or Cy5, respectively. Temperature was kept at 20°C using the in-built peltier system. Supplementary Fig. 11a depicts the spectra of the individually labelled PA nanofibres, showing significant overlap between Cy3 emission (donor) and Cy5 excitation (acceptor). On an independent measurement, PA nanofibres labelled with both Cy3 (1 mol%) and Cy5 (0, 0.1, 0.2 and 0.5 mol%) were assembled in 10 mM HEPES buffer (pH 7.5, NaCl 150 mM) at a final PA concentration of 0.5 mM. Fluorescence-emission spectra of dual-labelled PAs were recorded at the excitation wavelength of Cy3 520 nm, at which direct excitation of Cy5 is negligible. Supplementary Fig. 11b shows considerable energy transfer by FRET, which increases monotonically with acceptor concentration, providing evidence for co-assembly of both Cy3- and Cy5-labelled PAs in the same nanofibres.

### Ensemble molecular exchange kinetics

For molecular exchange kinetic experiments, two sets of PA nanofibres were pre-assembled from a mixture of non-labelled PAs and either Cy3 (0.5%) or Cy5 (0.5%). These independently labelled PA solutions were mixed at 1:1 ratio in a 50 μl quartz cuvette and fluorescence was followed over around 24 h (lag time ∼30 s). FRET ratio was determined by dividing Cy5 emission at 668 nm by Cy3 emission at 565 nm, at the Cy3 excitation wavelength (520 nm). The contribution of Cy3 emission at 668 nm was first subtracted from the Cy5 emission. At the beginning of the kinetics experiment, Cy3-PA and Cy5-PA are distributed in different nanofibres, being physically separated by distances much higher than the expected FRET radius^35^. Therefore, energy transfer at time zero should be negligible and, consequently, FRET ratio should be zero. A total PA concentration range of 0.5–5 mM was used. Kinetics was recorded at two different temperatures (20 or 37 °C).

### Sample preparation for microscopy

Glass microscope coverslips were cleaned by successively immersing in acetone, isopropanol and Milli-Q water. Bath sonication was performed for 10 min with each solvent, followed by drying under N_2_ flow. The glass coverslips were then etched with a fresh Piranha solution (3:1 v/v H_2_SO_4_ (98%):H_2_O_2_ (30%)) for 30 min. To finish the cleaning procedure, the slides were washed thoroughly with Milli-Q water and rinsed with acetone before drying under N_2_ flow. To determine the labelled PA distribution along the nanofibres length, single fibres should be immobilized on a glass surface, and remain still during STORM acquisition. A flow chamber was assembled using a glass slide and a clean coverslip separated by double-sided tape. PA nanofibres were immobilized by adsorption onto the surface of the clean coverslip by flushing diluted PA solutions at high ionic strength (NaCl 1 M). After incubation for 1 min, the unbound nanofibres were washed out of the chamber by flushing with HEPES buffer (2 × ) followed by STORM buffer (2 × ). STORM buffer is composed by 50 mM HEPES buffer (pH=8.5), 1 M NaCl, an oxygen-scavenging system (0.5 mg ml^−1^ glucose oxidase, 40 μg ml^−1^ catalase, 5 wt% glucose) and 200 mM 2-aminoethanethiol. The high ionic strength conditions were used to screen the electrostatic repulsion between highly charged PA nanofibres and the glass surface. However, repulsion between PA nanofibres is also reduced, causing fibre bundling. To avoid bundling, PA solutions were rapidly diluted at low ionic strength (1 μM). After a final (10 × ) dilution step in NaCl 1M, nanofibres were immediately attached on the glass coverslip. At this concentration isolated nanofibres are mainly observed in the microscopic mounting.

### Stochastic optical reconstruction microscopy

STORM images were acquired using a Nikon N-STORM system configured for total internal reflection fluorescence imaging. Excitation inclination was tuned to maximize the signal-to-noise ratio of the glass-absorbed fibres. Cy3- and Cy5-labelled samples were illuminated by 561 and 647 nm laser lines. No activation ultraviolet light was used. Fluorescence was collected by means of a Nikon 100 × , 1.4NA oil immersion objective and passed through a quad-band pass dichroic filter (97335 Nikon). All time-lapses were recorded onto a 64 × 64 pixel region (pixel size 0.17 μm) of an EMCCD camera (ixon3, Andor). For each channel, 20,000 frames were sequentially acquired. STORM movies were analysed with the STORM module of the NIS Elements Nikon software.

### Molecular imaging of fibres during exchange

PA nanofibres were separately labelled with either PA-Cy3 (5%) or PA-Cy5 (5%) at 0.5 mM in 10 mM HEPES buffer (pH 7.5, NaCl 150 mM). Subsequently, 250 μl-aliquots of each solution were mixed in a microcentrifuge tube and gently shaken at 37 °C for different amounts of time. At predefined time points, 2 μl of the solution was withdrawn, diluted and adsorbed on a glass surface as described above. We verified that once adsorbed on the slide, fibres are not able anymore to considerably exchange monomers with the solution in the time frame of the experiment. STORM imaging of Cy5 and Cy3 channels was performed sequentially. Fibres that are initially labelled only with Cy3 and incorporate Cy5 monomers over time were selected observing the fluorescence intensity in the low-resolution images. Original single-colour nanofibres were also observed before mixing as a control.

### Image analysis

During STORM imaging, the Nikon software generates a list of localizations by 2D Gaussian fitting of blinking chromophores in the acquired movie of conventional microscopic images. This localizations list was subsequently analysed with our custom-made Matlab scripts, as described in detail elsewhere^19^. Briefly, a first script uses a density-based clustering algorithm to automatically identify the fibres in the image and to remove the background. Examples of output localization maps are shown in Figs 3a,b and 5a. A second script is run on the well-reconstructed fibres obtained in the Cy3-channel to find the fibre backbone. The lower localization density obtained in the Cy5-channel for shorter time periods in the molecular exchange experiment is not enough to fully reconstruct the nanofibres. The polymer backbone coordinates are used to obtain structural information. Next, histograms of localization density profiles along the backbone are generated for both channels, as shown in Figs 3c,d and 5b. The spatial autocorrelation (that is, the average correlation between the localization density at one backbone point in the fibre and the density at another backbone point, as a function of the distance between these points) was also computed using the Matlab-function xcov with unbiased normalization. The spatial cross-correlation of the two channels was calculated in a similar manner. Averaging over multiple fibres is required to obtain accurate correlation decay graphs, such as depicted in Figs 3e,f and 6. Investigation of the average correlation decays yields the distribution of dyes in a fibre, without interference from the stochastic fluctuations inherent to the technique.

## Additional information

**How to cite this article:** da Silva, R. M. P. *et al*. Super-resolution microscopy reveals structural diversity in molecular exchange among peptide amphiphile nanofibres. *Nat. Commun.* 7:11561 doi: 10.1038/ncomms11561 (2016).

## Supplementary Material

Supplementary Information Supplementary Figures 1-11
