# Supplementary material for: Super-resolution microscopy reveals structural diversity in molecular exchange among peptide amphiphile nanofibres
Source: Nat Commun. 2016 May 19;7:11561. doi: 10.1038/ncomms11561 (PMC4874009; doi:10.1038/ncomms11561)
Supplement: Supplementary Information — Supplementary Figures 1-11 [file ncomms11561-s1.pdf]

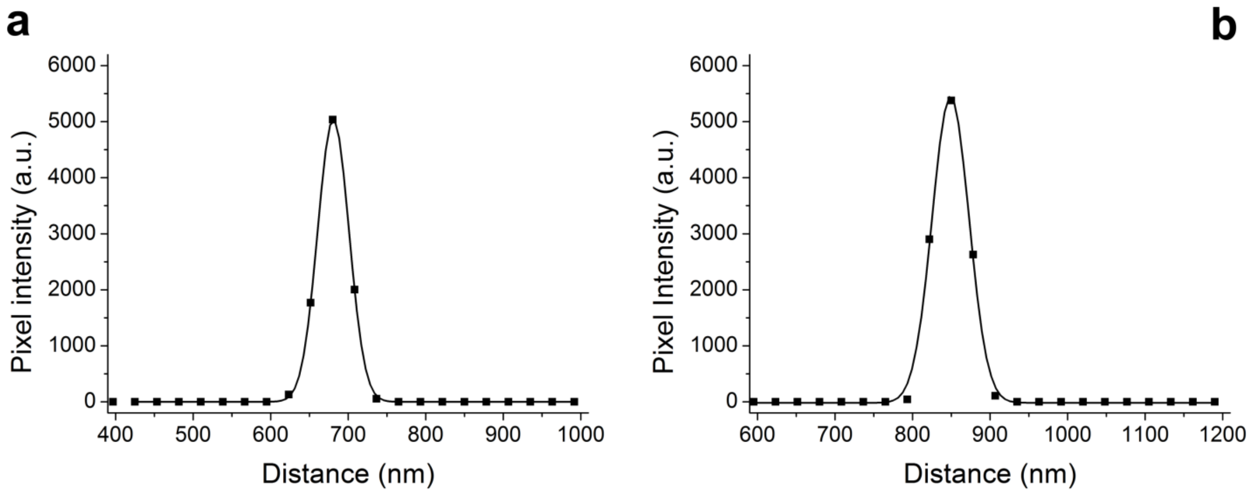

Supplementary Figure 1 | STORM resolution. Intensity profiles along the cross-section of PA nanofibres depicted in Supplementary Fig. 3b, in the regions marked with a blue arrow. Single fibres were picked choosing the thinner filaments visible in the picture. A Gaussian fit revealed an image resolution of around 50 nm, as measured by the FWMH. (**a**) Histogram corresponding to arrow 1 of Supplementary Fig. 3b (FWMH = 47 nm). (**b**) Histogram corresponding to arrow 2 of Supplementary Fig. 3b (FWMH = 53 nm).

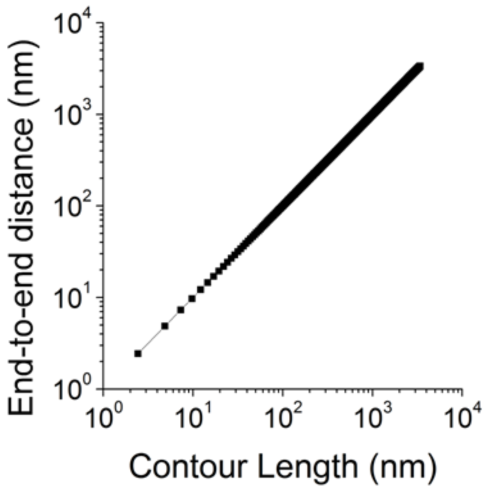

Supplementary Figure 2 | Scaling properties of PA nanofibres. Mechanical properties of fibres were estimated fitting nanofibres backbone. Scaling behavior was determined averaging the 30 longest fibres in the full dataset. A constant slope close to unity reveals a micrometer range persistence length, consistent with a rigid rod behavior for the entire nanofibre length scale.

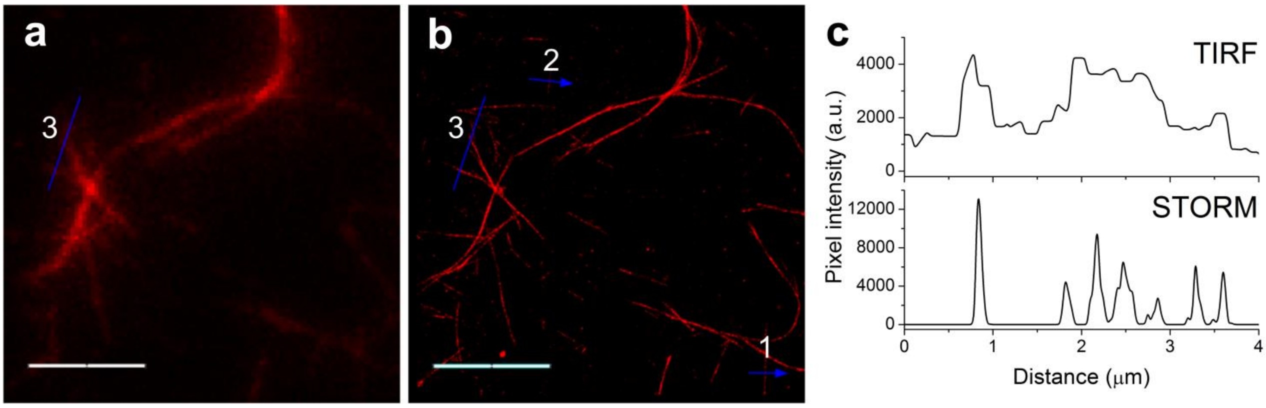

Supplementary Figure 3 | Super-resolution fluorescence microscopy image (b) revealing fine details which otherwise could not be detected using a diffraction limited technique (a). (**a**) Fluorescence microscopy images of PA nanofibres co-assembled with 5% of Cy5-labeled PA adsorbed at high density in a glass surface. At this surface density, overlaying and bundling of different nanofibres do not allow to resolve single structures using total internal reflection fluorescence (TIRF) microscopy. This wide field technique is diffraction limited and the resolution is around 250 nm. (**b**) Stochastic Optical Reconstruction Microscopy (STORM) image of the same area revealing sub-diffraction details. The photoswitchable behavior of the dyes allows single molecule detection with a localization precision of labeled PA molecules of around 50 nm (see Supplementary Fig. 1 for intensity profiles along arrows 1 and 2). (**c**) Intensity profiles along the straight line 3, showing the ability of STORM to reveal the presence of 7 fibres which could not be distinguished in low resolution TIRF microscopy. Scale bars correspond to 5 µm.

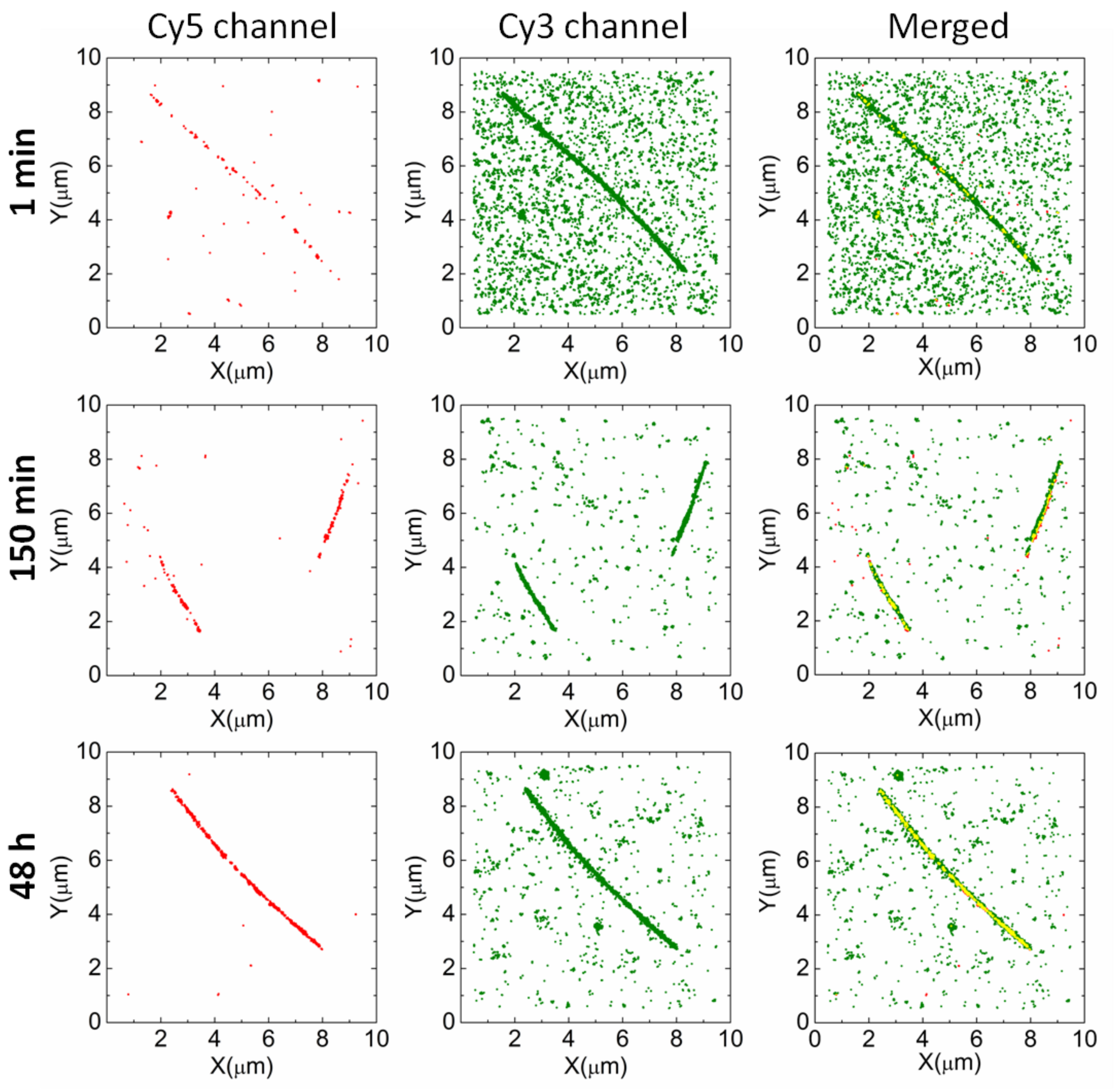

Supplementary Figure 4 | Molecular exchange kinetics. Time course STORM images show the progressive incorporation of Cy5 (red) labeled molecules on initially Cy3 (green) labeled fibres. PA nanofibres were premixed (labeled) with either Cy3 or Cy5 at 5 mol% and reconstituted in aqueous saline HEPES buffer solution (pH 7.5, NaCl 0.150M). Equal volumes of each solution of labeled PA nanofibres were mixed (t=0). STORM images were acquired after immobilization of nanofibres in glass coverslips at different time points. For the initial time points, fibres were selected based on the higher intensity on the green (561 nm laser) than on the red channel (647 nm), as observed by diffraction limited TIRF mode. At 48h, fibres presented similar intensities in both channels.

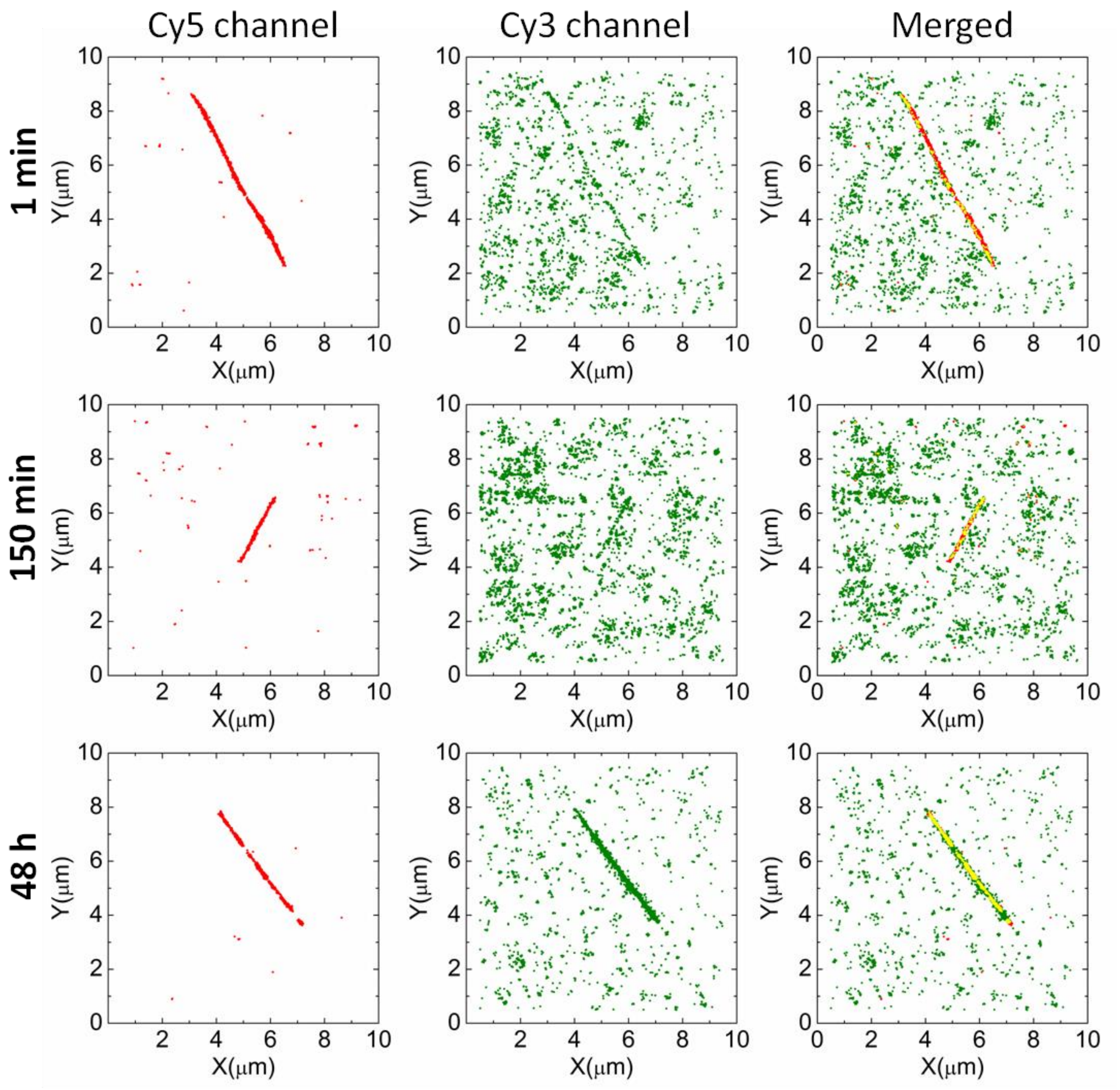

Supplementary Figure 5 | Molecular exchange kinetics. Time course STORM images showing the progressive incorporation of Cy3 (green) labeled molecules on initially Cy5 (red) labeled fibres. For the initial time points, fibres were selected based on the higher intensity on the red (647 nm laser) than on the green channel (561 nm), as observed by diffraction limited TIRF mode. At 48h, fibres presented similar intensities in both channels.

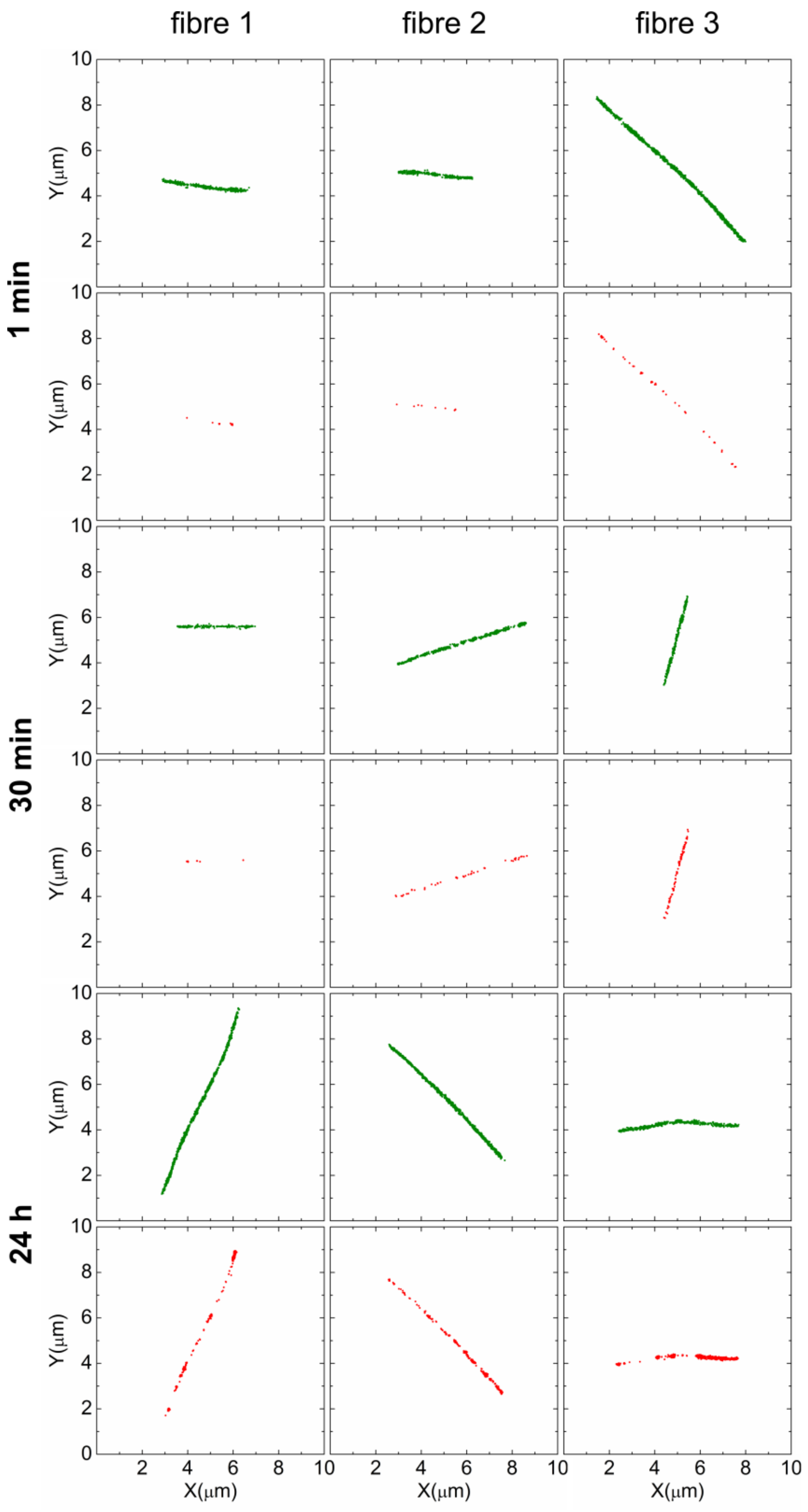

Supplementary Figure 6 | Heterogeneous molecular exchange kinetics. Time course STORM images showing that the progressive incorporation of Cy5 (red) labeled molecules on initially Cy3 (green) labeled fibres is not a homogenous process, with locally different molecular exchange rates. Three fibre examples for each time point.

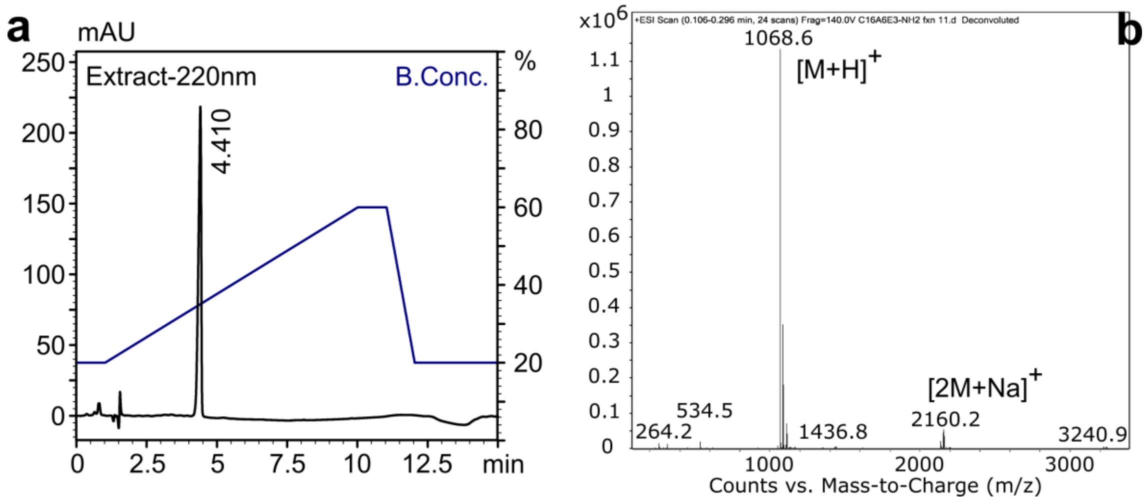

Supplementary Figure 7 | Purity of non-labeled PA. (**a**) Analytical HPLC and (**b**) mass spectrum of non-labeled PA [expected m/z: 1068.61 (100.0%), 1069.61 (54.6%), 1070.61 (19.4%)].

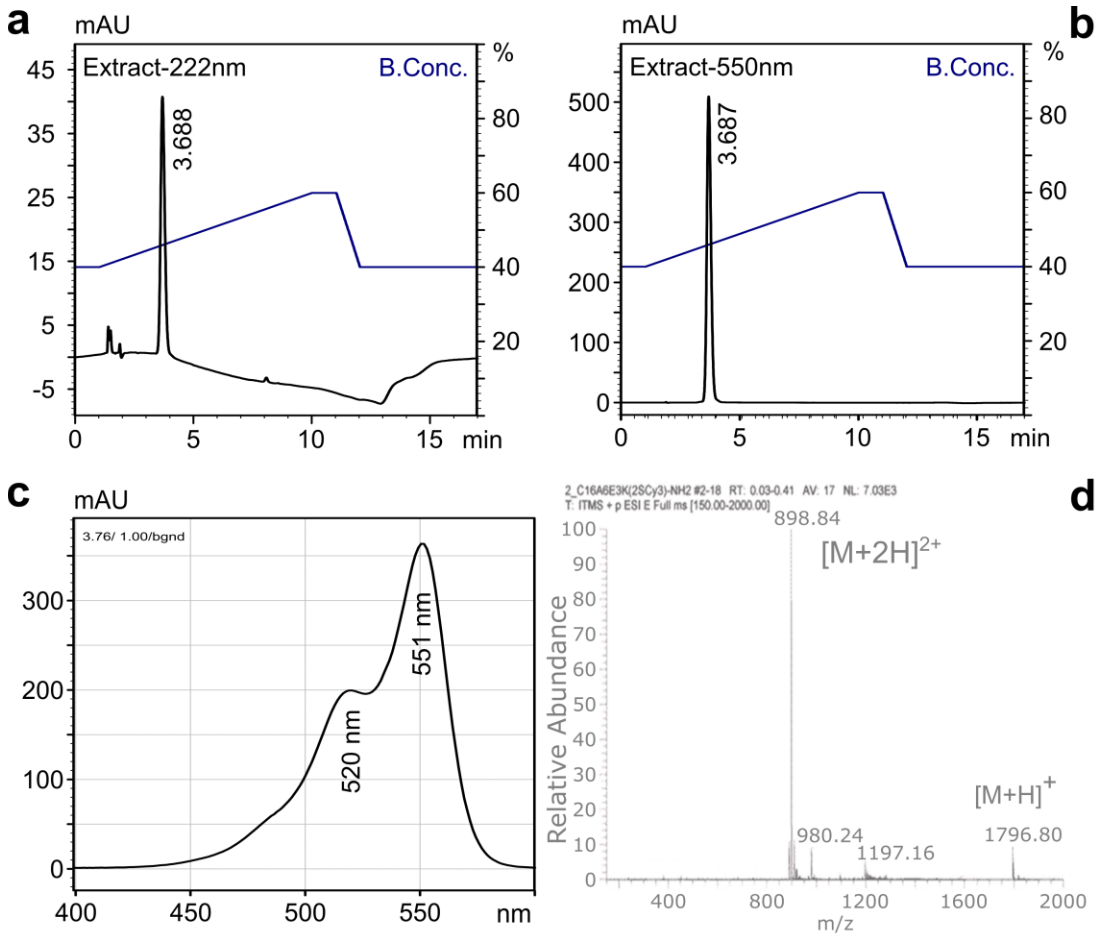

Supplementary Figure 8 | Purity of Cy3-labeled PA. (**a**) Analytical HPLC traces at 222 nm (peptide absorption region) and (**b**) at 550 nm (Cy3 absorption maximum). (**c**) The UV-Vis spectrum taken at 3.8 min shows the characteristic absorption of coupled Cy3 dyes. (**d**) The mass spectrum of Cy3-labeled PA [expected m/z: 1794.88 (100.0%), 1795.89 (94.3%), 1796.89 (50.5%), 1797.89 (20.6%)].

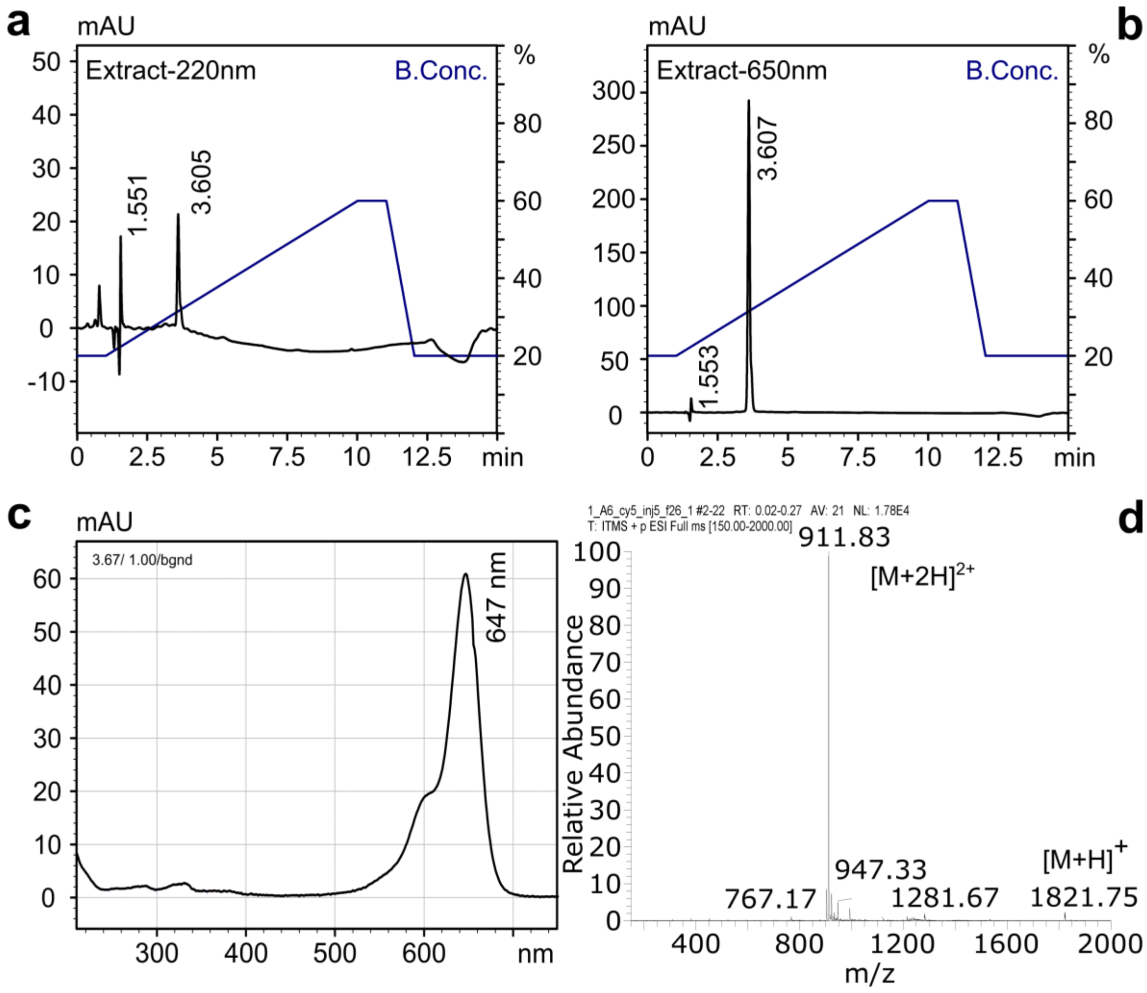

Supplementary Figure 9. Purity of Cy5-labeled PA. (**a**) Analytical HPLC traces at 220 nm (peptide absorption region) and (**b**) at 650 nm (Cy5 absorption maximum). (**c**) The UV-Vis spectrum taken at 3.7 min shows the characteristic absorption of coupled Cy5 dyes. (**d**) The mass spectrum of Cy5-labeled PA [expected m/z: 1821.90 (100.0%), 1820.90 (96.8%), 1822.90 (53.5%), 1823.91 (18.6%)].

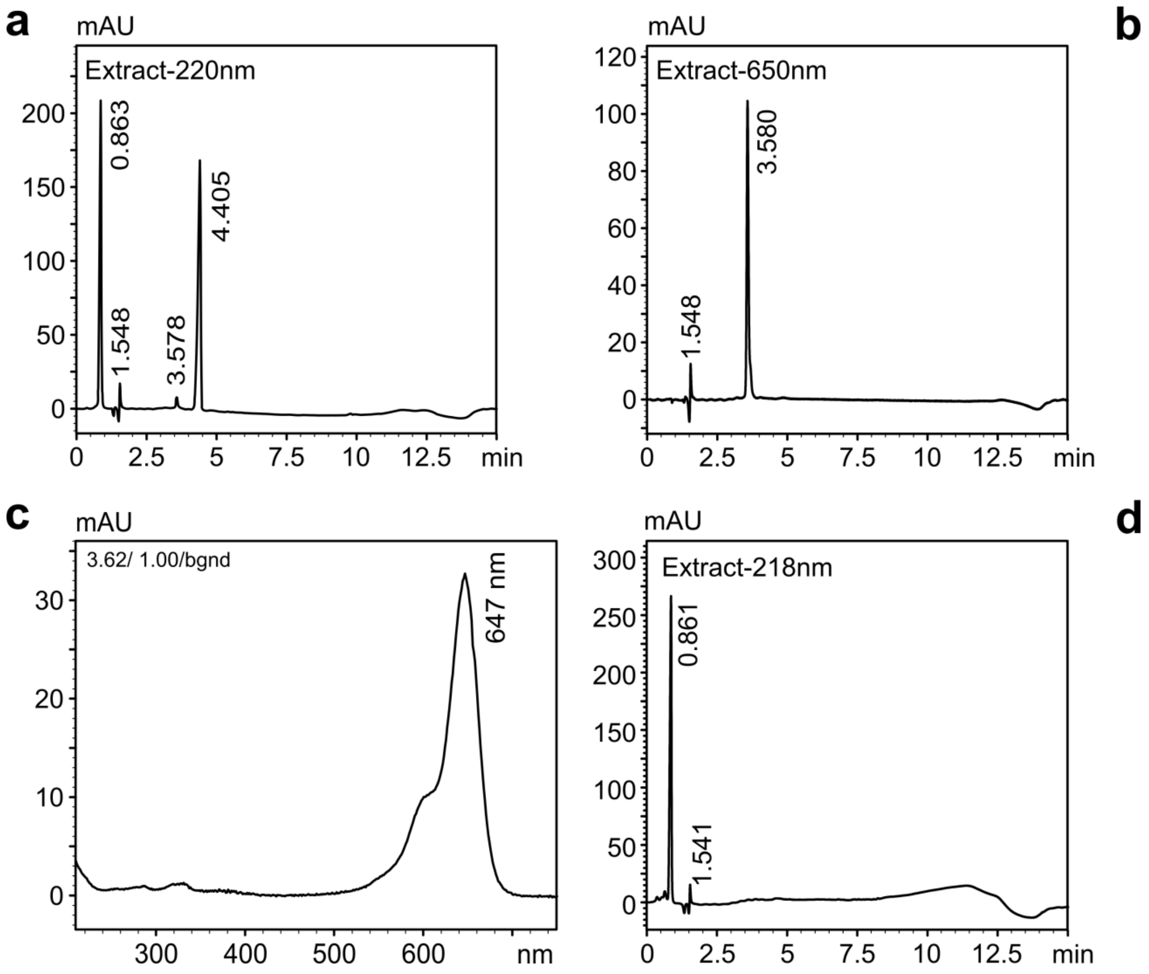

Supplementary Figure 10 | Analytical HPLC of Cy5 labeled PA co-assembled with non-labeled PA (premixture). The harsh acidic conditions (dissolution in TFA) used to create a homogenous molecular mixture of different PAs were checked by analytical HPLC to rule out potential degradation of the original molecules. (**a**) HPLC UV trace at 220 nm showing both Cy5-labeled (retention time 3.58 min) and non-labeled PA (4.40 min). The peak at 0.86 min corresponds to background HEPES buffer used to reconstitute the sample. (**b**) HPLC trace at the Cy5 wavelength at maximum absorption intensity, showing the Cy5 labeled PA without traces of other Cy5 coupled species. (**c**) UV-Vis spectrum at retention time of 3.58 min, showing the expected absorption spectrum for Cy5 labeled PA. (**d**) Blank UV trace at 220 nm, confirming that product with retention time 0.86 min origins from the HEPES buffer used to reconstitute the sample.

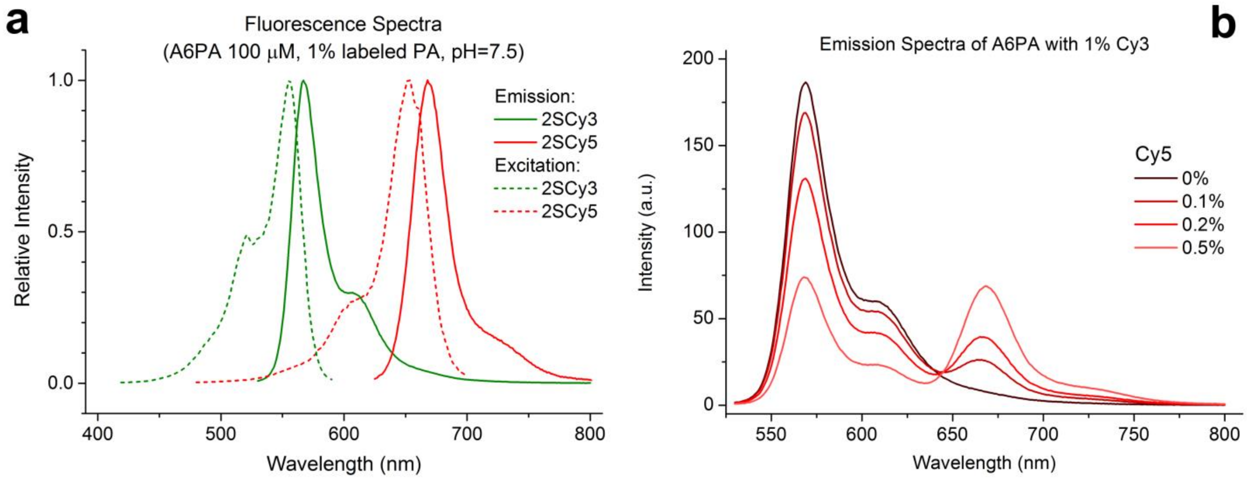

Supplementary Figure 11 | Förster resonance energy transfer (FRET) between Cy3 and Cy5 labeled PAs. (**a**) The normalized Fluorescence excitation and emission spectra of either Cy3 or Cy5 labeled PAs show a high degree of over-lapping between Cy3 emission (Donor) and Cy5 excitation (Acceptor). Labeled PAs were premixed at 1 mol% and reconstituted in HEPES buffer (pH 7.5). (**b**) A decrease of Cy3 emission intensity is observed as Cy5 content increases in premixed nanofibres. A consequent increase in Cy5 emission due to FRET is observed. Contribution from direct excitation of Cy5 is negligible at the wavelength (520 nm) used to excite Cy3 fluorophores.
